# Male delayed orgasm and anorgasmia: a practical guide for sexual medicine providers

**DOI:** 10.1038/s41443-023-00692-7

**Published:** 2023-04-15

**Authors:** Vi Nguyen, Isabella Dolendo, Maria Uloko, Tung-Chin Hsieh, Darshan Patel

**Affiliations:** Department of Urology, University of California, San Diego, CA USA

**Keywords:** Reproductive signs and symptoms, Sexual dysfunction

## Abstract

Delayed orgasm (DO) is defined as increased latency of orgasm despite adequate sexual stimulation and desire. Anorgasmia (AO) is characterized as the absence of orgasm. Etiologies of DO/AO include medication-induced, psychogenic, endocrine, and genitopelvic dysesthesia. Given the multifactorial complex nature of this disorder, a thorough history and physical examination represent the most critical components of patient evaluation in the clinical setting. Treating DO/AO can be challenging due to the lack of standardized FDA-approved pharmacotherapies. There is no standardized treatment plan for DO/AO, though common treatments plans are often multidisciplinary and may include adjustment of offending medications and sex therapy. In this review, we summarize the etiology, diagnosis, and treatment of DO/AO.

## Introduction

Male sexual function involves complex interactions among psychosocial, neurogenic, vascular, and endocrine factors that contribute to initiation of erection culminating in ejaculation and orgasm [1, 2]. Male sexual dysfunction occurs with disruption of any of these processes and encompasses decreased libido, erectile dysfunction (ED), ejaculation disorders, and orgasmic dysfunction [3]. ED receives the most attention and concordantly is the most well-studied and understood by patients and providers [4]. Compared to ED where reliable and well-studied treatments are available, there remains a paucity of standardized treatments for men with ejaculatory and/or orgasmic dysfunction. There are extensive well-studied treatments for ED including multiple phosphodiesterase-5 inhibitors, vacuum devices, self-injectable drugs, and penile prosthesis; meanwhile, there are no FDA approved pharmacotherapies or invasive non-pharmacological interventions approved for delayed orgasm (DO) [5, 6]. However, orgasm and ejaculation represent fundamental aspects of male sexual function, and ejaculatory and orgasmic dysfunction are highly prevalent and tremendously impact on quality of life [7–9].

Male orgasmic dysfunction can be dichotomized as two ends of a spectrum: premature ejaculation (PE) versus DO and anorgasmia (AO) [10, 11]. Though ejaculation and orgasm are distinct events, they occur simultaneously in men and often are used interchangeably in the literature when discussing delayed ejaculation (DE) vs. DO [6]. In this review, we specifically focus on summarizing the current knowledge regarding the epidemiology, pathophysiology, diagnosis, and treatment of DO/AO. Recently, the American Urological Association (AUA) released the Disorders of Ejaculation guidelines that we will also highlight [6].

## Methodology

In this narrative review, the PubMed database was used to identify articles using the search terms: male sexual dysfunction, male delayed orgasm, delayed ejaculation, anorgasmia, male orgasm, ejaculation. There was no limitation on date range. Clinical trials, case studies, abstracts, review articles, and questionnaire-based surveys were included. Only studies published in English were considered.

## Anatomy and physiology of male ejaculation and orgasm

It is important to realize that ejaculation and orgasm are two separate processes as orgasm can occur without ejaculation. However, as aforementioned, studies surrounding delay in these processes often use the terms DE and DO interchangeably.

Male ejaculation can be divided into two distinct phases: emission and expulsion [12]. The first phase of emission is characterized by passage of seminal fluid from the prostate, seminal vesicles, and vas deferens into the posterior urethra [13]. This occurs concomitantly with contraction of the internal urethral sphincter, which closes the bladder neck to prevent retrograde passage of semen [13]. This process is dependent on smooth muscle contraction and autonomic innervation from the pelvic plexus [14]. Stimuli from sensory receptors at the glans integrate at the spinal level to stimulate emission [14]. Expulsion, also known as antegrade ejaculation, is the second phase of ejaculation and is characterized by the passage of seminal fluid from the posterior urethra to the external urethral meatus [15]. This is regulated by contractions of striated pelvic floor muscles [15–17].

Male orgasm occurs secondary to pressure buildup within the posterior urethra due to closure of the bladder neck and external urethral sphincter and contraction of the periurethral musculature [13].

The anatomy of orgasm has been delineated by several functional neuroimaging studies; a meta-analysis demonstrated that in both heterosexual and homosexual men, sites of cortical activation include the lateral occipitotemporal, inferotemporal, parietal, orbitofrontal, medial prefrontal, insular, anterior cingulate, and frontal premotor cortices [18]. Likewise, positron emission tomography (PET) studies concordantly demonstrate increased activity in the occipitotemporal lobes, anterior cingulate, insular cortices, bilateral substantia nigra, right pons, left dentate cerebellar nucleus, and left lateral midbrain during orgasm [19]. Conversely, there is a decrease in the regional cerebral blood flow across the prefrontal cortex during ejaculation [18]. Flannigan et al. observed increased activation in the right fusiform gyrus among men with DO compared to controls; using the Allen Atlas of Human Brain Expression, corresponding neurotransmitter receptors to this region include adenosine receptors, muscarinic and nicotinic cholinergic receptors, cannabinoid receptors, and dopamine receptors [20]. These receptors serve as potential targets for novel pharmacotherapies.

## Definition of DO/AO

Although most definitions are similar, there is no standardized definition for DO. The AUA guidelines on disorders of ejaculation recognize that there are multiple terms to refer to delays in ejaculations and/or orgasm including DE and DO [6]. Though it is acknowledged that ejaculation and orgasm are separate entities, the term DE is often used to refer to both difficulties with ejaculation and orgasm. Additionally, the AUA guidelines consider AO to be “the condition in which sexual climax cannot be reached via any means of stimulation” [6].

The Diagnostic and Statistical Manual of Mental Disorders 5th Edition (DSM-V) states that “distressing difficulties with orgasm in men would be considered under delayed ejaculation,” which is defined as a marked delay in ejaculation or a marked infrequency of absence of ejaculation on 75–100% of all occasions of partnered sexual activity without the individual desiring delay, persisting for at least 6 months, and causing significant distress to the individual [11]. DSM-V does not provide a definition for AO [11].

The International Classification of Diseases (ICD)-11 defines DE as an inability to achieve ejaculation or an excessive or increased latency of ejaculation, despite adequate sexual stimulation and the desire to ejaculate [21]. The ICD-11 defines AO as “the absence or marked infrequency of the orgasm experience or markedly diminished intensity of orgasmic sensations,” though states that AO in men would be encompassed in the diagnosis of DE [21]. For both DO and AO, these patterns of orgasmic dysfunction have occurred episodically or persistently over a period of at least several months and is associated with clinically significant distress [21]. AO is often considered to be the most extreme presentation of DO and thus has similar etiology and treatment options.

DO can also be further categorized as lifelong/primary (present since first sexual encounter) versus acquired/secondary (prior normal orgasm able to be achieved) and generalized (always present) versus situational (only present due to certain stimuli, with certain partners, or in certain situations) [22]. The Third International Consultation on Sexual Medicine defines the time threshold for DO based on studies examining mean intravaginal ejaculation latency time (IELT; defined as the time between the start of vaginal intromission and the start of intravaginal ejaculation) [23]. Waldinger et al. measured stopwatch-assessed IELT among men from the Netherlands, United Kingdom, Spain, Turkey, and the United States and determined that median IELT is 5.4 min (range: 0.55–44.1 min) [24]. Thus, it has been extrapolated that IELT for DO would be 2 standard deviations above, equivalent to an estimated 20–25 min [22].

## Epidemiology

Kinsey et al. found that on 15 out of 10,000 participants had primary AO [25] while Nathan et al. found the prevalence of inhibited male orgasm to be 5% [26]. DO and DE are used interchangeably in the literature, the true prevalence of AO and DO is difficult to extrapolate since difficulties with orgasm and ejaculation are often not separated in studies. Corona et al. evaluated 2652 men and asked patients about difficulty with ejaculation and climax. They observed a 7.3% (*n* = 194) prevalence of DE and/or DO [10]. In a separate study, the same author group evaluated the prevalence of sexual dysfunction among 2437 men with mean age 51.9 years and reported a 4.4% rate of DE and/or DO [27]. However, it is postulated that the prevalence of DO is likely higher as embarrassment may preclude men from seeking treatment or discussing this condition with their providers [28].

## Pathophysiology of DO

Several etiologies have been identified in the pathophysiology of DO, owing to the multifactorial, complex nature of the disorder. The most common etiologies include selective serotonin reuptake inhibitors (SSRI; 42%), psychogenic (28%), low testosterone (T; 21%), abnormal penile sensation (7%), and penile hyperstimulation (2%) [29].

### Endocrinopathies

Given the fundamental role of hormonal regulation in the physiology of orgasm, multiple endocrinopathies have been identified in patients with DO. Corona et al. compared T levels among 2437 men (mean age 51.9 ± 13.0 years) with PE or DO versus those without ejaculatory dysfunction and demonstrated lower total and free T levels among patients with DO [27]. Patients with DO had higher prevalence of testosterone deficiency when compared to patients with PE (26 vs. 12%, respectively) [27]. The varied prevalence of testosterone deficiency among patients with PE and DO compared to the control group remained statistically significant even when controlling for age (HR = 0.75 [0.57–0.99] and 1.83 [1.14–3.94], respectively; both *p* < 0.05) [27].

Hyperprolactinemia also leads to DO as increased levels of prolactin (PRL) result in suppression of T production [30]. Hyperprolactinemia can be defined as mild (>420 mU/L or 20 ng/mL) versus severe (>735 mU/L or 35 ng/mL) [30]. It has been shown that PRL levels progressively increase when comparing men with PE versus no ejaculatory dysfunction versus DO respectively [10].

Likewise, aberrant thyroid stimulating hormone (TSH) levels have been associated with orgasmic dysfunction. Hyperthyroid patients often experience PE whereas hypothyroid patients often experience DO. A multicenter prospective study of 48 men (*n* = 34 hyperthyroid, *n* = 14 hypothyroid) demonstrated that IELT significantly improved after normalization of TSH levels [31]. Among hyperthyroid men, IELT doubled from 2.4 min to 4.0 min after treatment, whereas IELT significantly decreased from 22 to 7 min in hypothyroid men [31].

### Medications

The most common medications implicated in the pathogenesis of DO include antidepressants and antipsychotics (Table 1) [32–38]. The underlying mechanism of SSRI-induced DO is that serotoninergic inputs from the dorsal raphe nucleus stimulate PRL-releasing factors in the paraventricular nucleus [39], resulting in hyperprolactinemia and subsequent suppression of T production as described above [14]. Furthermore, SSRIs are associated with a twofold risk of lower libido [40]. The rate of orgasmic dysfunction at 8 weeks following initiation of antidepressant therapy are as follows: escitalopram (30%), bupropion (15%), placebo (9%) [41].

**Table 1:** Medications associated with delayed orgasm or anorgasmia.

Drug	Drug Class	Reported Prevalence of DO/AO
Sertraline	SSRI	11–67% [32]
Citalopram	SSRI	2–63% [32]
Fluvoxamine	SSRI	9–54% [32]
Fluoxetine	SSRI	24–75% [32–34]
Paroxetine	SSRI	20–54% [32, 33]
Escitalopram	SSRI	4–30% [32, 35]
Venlafaxine	SNRI	20–62% [32, 33]
Duloxetine	SNRI	33% [36]
Bupropion	Norepinephrine and dopamine reuptake inhibitor	7–22% [32]
Clomipramine	TCA	15–92% [32]
Imipramine	TCA	5–21% [32, 35]
Amitriptyline	TCA	10% [32]
Phenelzine	MAOI	11–40% [34, 35]
Tranylcypromine	MAOI	40% [34]
Reboxetine	Norepinephrine reuptake inhibitor	5–10% [34]
Haloperidol	Antipsychotic	40–60% [35]
Thioridazine	Antipsychotic	40–60% [35]
Methadone	Opioid	14–81% [37, 38]

*SSRI* Selective serotonin reuptake inhibitor, *SNRI* Serotonin norepinephrine reuptake inhibitor, *TCA* Tricyclic antidepressant, *MAO*I Monoamine oxidase inhibitor, *DO* Delayed Orgasm, *AO* Anorgasmia.

### Psychogenic

Psychogenic DO results from feelings of fear, anxiety, hostility, relationship difficulties associated with sexual intercourse and encounters [42]. Common triggers include childhood sexual abuse, sexual trauma, repressive sexual education or religious beliefs, general anxiety, and history of being widowed or divorced [43].

One specific scenario that has been studied in patients with situational DO is timed intercourse for fertility treatment [44]. Byun et al. demonstrated a 42.8% (*n* = 188) and 5.92% (*n* = 26) rate of ED and DO respectively among 439 men undergoing timed intercourse. The Beck Anxiety Inventory, a standardized measure of anxiety level, was significantly higher among men with DO (*p* < 0.001).

### Penile hyperstimulation and sensation loss

Hyperstimulation of the penis is another factor that has been identified to contribute to DO. DO is significantly associated with higher masturbatory activity, decreased nighttime emissions, lower orgasm and intercourse satisfaction scores on the International Index of Erectile Function (IIEF) [45]. It is postulated that increased frequency of masturbation results in penile sensation loss which cascades into further increase of masturbation force. Consequently, this cycle results in DO during vaginal intercourse or orogenital stimulation as these processes are not able to replicate the necessary stimulation. Penile sensation loss has also been associated with increasing age [46].

### Pelvic surgery patients

Orgasmic dysfunction has been demonstrated among patients who undergo pelvic surgeries [47]. Haney et al. reviewed the current literature on the prevalence of orgasmic dysfunction post-pelvic surgery and reported the following AO (AO) rates: radical prostatectomy (5–70%); radical cystectomy (33–63%); colorectal surgeries (0–52%) [47]. Preservation of orgasmic function is directly correlated to intraoperative nerve sparing; 90.7, 82.1, and 60.8% of men who underwent bilateral (*n* = 273/301), unilateral (46/56), and non-nerve sparing (*n* = 31/51) robotic-assisted laparoscopic prostatectomy reported preservation of orgasmic function post-operatively (*p* < 0.001) [48].

## Patient evaluation and diagnosis of DO

Initial evaluation of a patient presenting with DO involves a thorough history and physical examination. This is the most important component of the patient’s evaluation. Critical components of the history intake include medical, surgical, psychiatric, sexual, social, and religious history. Medical history should include conditions associated with neuropathy, metabolic derangements, or trauma; surgical history should include the history of neurologic and pelvic procedures [49]. To date, no studies have identified an association between orgasmic dysfunction and recreational drug use, pornography use, or painful intercourse, but these still represent important components of the social history. Psychiatric history should delve into any stressors at home or work, history of infertility, sexual abuse or trauma, repressive sexual education or religious beliefs, and history of being widowed or divorced [22, 43].

Questioning regarding onset and duration of DO can also enable the clinician to decipher between lifelong/primary versus acquired/secondary DO and generalized versus situational DO [6]. The patient’s medication list should also be reviewed [6].

General physical examination is also performed to evaluate for any confounding organic conditions including metabolic disorders (obesity), decreased serum T (minimal body hair, muscular atrophy), and prior injuries (scars) [50–52]. Genitourinary examination should assess for undescended or solitary testicle as well as testicular atrophy [6].

Hormonal evaluation serves as the basis for laboratory testing when evaluating a patient with DO. The mainstay of hormonal laboratory testing is total and calculated bioavailable/free T, TSH, vitamin D, estradiol, and PRL [53, 54]. Baseline labs such as a basic metabolic panel (BMP), human immunodeficiency virus (HIV) testing, Hemoglobin A1c may also be assessed to evaluate for other underlying abnormalities as indicated by the patient’s history [51, 54, 55].

Adjunctive testing includes biothesiometry or pudendal somatosensory-evoked potential (SSEP) to evaluate for loss of penile sensitivity, sympathetic skin testing to assess sympathetic efferent flow to genital skin, and sacral reflex art testing to examine the motor and sensory branches of the pudendal nerves and the S2-4 nerve roots [22]. However, adjunctive testing is not routinely clinically indicated.

**Fig. 1:**
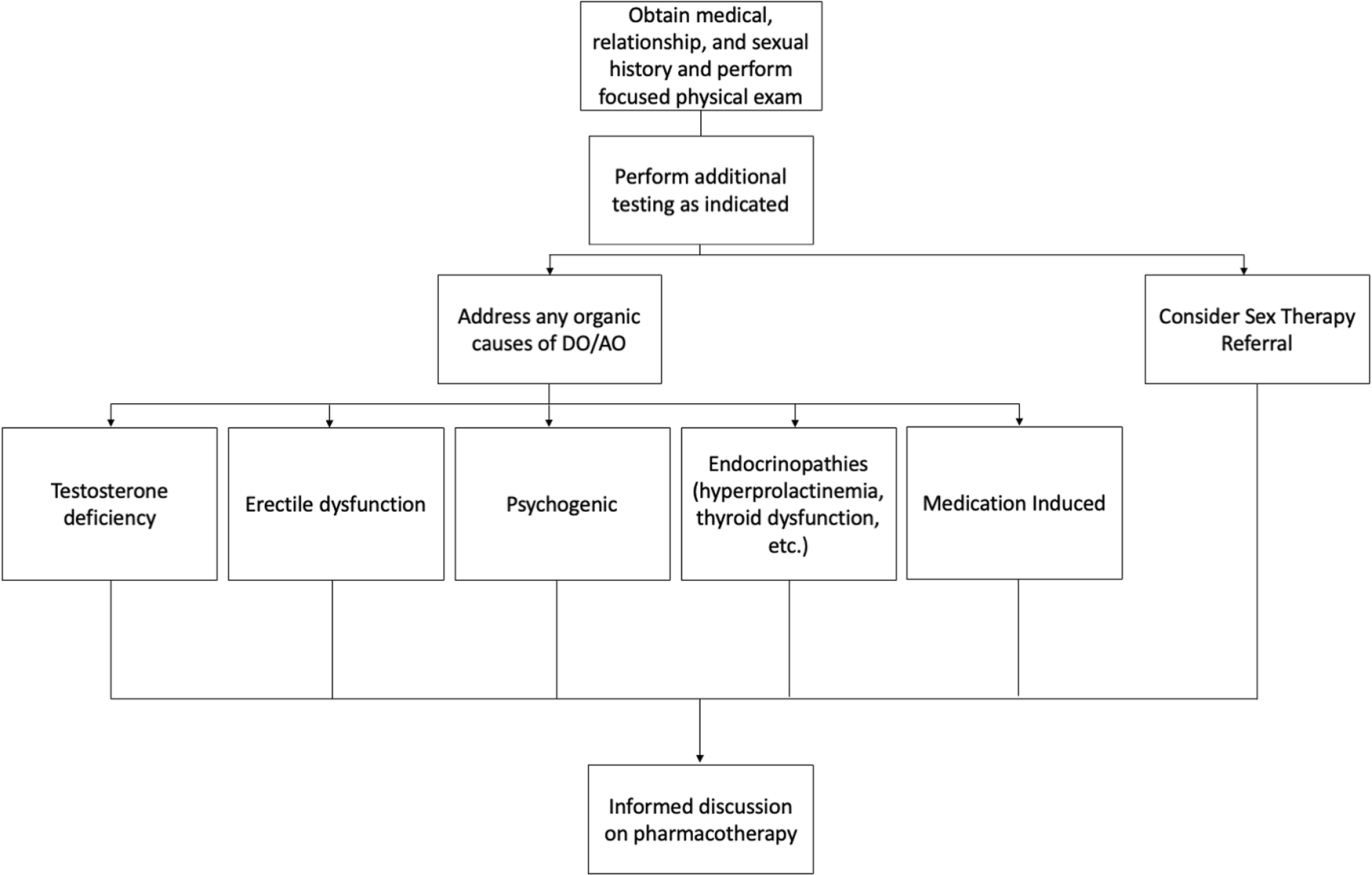
Proposed algorithm to approaching the patient who presents with DO/AO.

A proposed algorithm summarizing the evaluation of patients presenting with DO/AO is summarized in Fig. 1.

## Treatment of DO

There is no standardized treatment plan for DO. Treatment plans are often multidisciplinary, involving urologists, primary care providers and mental health professionals to adequately address biopsychosocial factors. Before initiation of treatment, patients should be evaluated for organic causes. If DO is associated with SSRIs or other medications, patients should discuss medication adjustment with the prescribing physician [56]. As previously discussed, there are certain medications that have been associated with DO/AO (Table 1). Offending medications may be decreased or stopped altogether. An alternative medication with lower risk of orgasmic dysfunction may be recommended as a substitute. Additionally, medications with a shorter half-life may also be preferable if a patient is experiencing sexual side effects [6].

Testosterone replacement is indicated for men with testosterone deficiency in accordance with available guidelines [57, 58]. In men with DO as their primary symptom of testosterone deficiency, we will consider a trial of testosterone therapy (injectables, topical, etc.). Anecdotally, we have used short-acting testosterone formulations that have rapid peak serum levels (intranasal testosterone gel) on demand (administered 1 h prior to sexual activity) in men with isolated DO and who do not desire to be on long-term testosterone therapy. ED can contribute to DO and thus should be treated with phosphodiesterase-5 inhibitors, self-injections, and/or vacuum devices [56]. In patients with hyperprolactinemia or thyroid dysfunction, appropriate referrals should be made.

### Sex therapy

Psychosocial evaluation with a sex therapist is recommended for all patients [57]. Sex therapy may be used as a monotherapy although it is often used in conjunction with other forms of treatment. Therapy can include sex education, cognitive-behavioral therapy (CBT), couples therapy, psychodynamic exploration, and mindfulness [59]. The suggested approach differs depending on the root cause of the patient’s DO. Insufficient stimulation can occur due to diminished penile sensation both with a partner and during self-stimulation or due to a psychogenic etiology which is more likely to occur only with a partner. A sex therapy approach for these patients involves enhancing psychological arousal by using a vibrator or vigorous pelvic thrusting and addressing psychological factors that may be contributing to DO/AO [6]. Other patients prefer self-stimulation that impacts their ability to orgasm with a partner. For these patients, therapy is anchored on shifting focus from himself to his ability to receive pleasure from his partner [6]. For a subset of patients who experience a discrepancy between stimulation from a partner and personal masturbation techniques, masturbatory retraining can allow the patient to condition himself to respond to certain sexual stimuli that could ultimately be replicated with a partner [6].

Psychological intervention can also identify and address a patient’s psychological conflict with ejaculation that could stem from a variety of sources including discord between partners, fear that ejaculation will impregnate their partner, strife with relinquishing control, or phobia of success [60, 61]. Counselors may help the patient identify interpersonal factors or psychological barriers that are negatively impacting sexual function such as communication issues between partners or anxiety related to sexual activity [62]. Therapy should also aim to destigmatize the condition [62].

Exact rates of success are difficult to determine based on existing literature due to variability of therapy methods and lack of large-scale studies. Having a sex therapist available for referrals is an important consideration for providers since there can be issues with accessibility including long waiting lists and financial barriers since insurance coverage is variable. If such barriers to sex therapy exist, providers can begin by suggesting open discussions about sexual desires and preferences between the patient and their partner to facilitate treatment. Other low risk behavior modifications include erogenous zone stimulation, altering pressure and pace of penile stimulatory techniques, using vibrators, or incorporating roleplay. Modification should be based on the couple’s current sexual practice or positions, and the sexual desires and comfort levels of each partner [6].

### Penile vibratory stimulation

If the patient reports decreased penile sensation and/or biothesiometry reveals abnormal penile sensitivity, penile vibratory stimulation (PVS) can be incorporated. During this treatment, a vibrator is applied to the frenulum of the penis for up to 10 min [63]. A study of 36 men with AO found that 72% of men who underwent PVS had restoration of orgasm on at least some occasions and these lasted at 6 month follow up [63]. However, large double-blind placebo-controlled studies are lacking and still needed to elucidate the true effectiveness of PVS. Although the current evidence is not conclusive to support the routine use of PVS, the AUA guidelines state that PVS may be recommended for interested patients given the minimal risk [6].

### Pharmacotherapy

Though there are no drugs approved by the U.S. Food and Drug Administration (FDA) at this time due to the absence of conclusive data, several pharmacological therapies show promise in treatment of DO including cabergoline, bupropion, oxytocin, and recently amphetamine/dextroamphetamine (Adderall^TM^)^22^. A summary is provided in Table 2. Importantly, we should try to address any organic causes and/or sex therapy prior to or in conjunction with pharmacotherapy. Clinicians should work with prescribing providers to stop or decrease the dose of medications that may contribute to DO/AO [6].

**Table 2:** Summary table of pharmacotherapies for delayed orgasm or anorgasmia.

Drug	Dose	Mechanism of Action	Common Side Effects
Cabergoline	0.5 mg twice weekly	Dopamine receptor agonist	Nausea, dizziness, headache
Bupropion	150 mg daily	Norepinephrine and dopamine reuptake inhibitor	Palpitations, chest pain, agitation, blurred vision, urinary frequency, psychosis
Oxytocin	16-24 IU intranasally	Acts on peripheral oxytocin receptors or vasopressin receptors	Nasal discomfort, irritability, tiredness
Amphetamine/dextroamphetamine	5–20 mg 1-2 h before sexual activity	CNS stimulant	Insomnia, headache, dry mouth, tachycardia, hypertension, restlessness, irritability
Midodrine	5–30 mg daily	α-1 agonist	Piloerection, pruritis, dysuria, paresthesia
Imipramine	20–100 mg daily	α-1 agonist	Hypertension, nausea, weakness, dry mouth
Ephedrine	30–150 mg daily	α-1 agonist	Nausea, vomiting, insomnia, nervousness, tachycardia
Pseudoephedrine	30–240 mg daily	α-1 agonist	Nausea, headache, dry mouth, irregular heartbeat, hypertension
Amantadine	75–100 mg 2-3x daily Or 100–400 mg 1-2 h before sexual activity	NMDA receptor antagonist	Orthostatic hypotension, syncope, peripheral edema, dizziness, delusions, hallucinations, paranoia, dry mouth, constipation
Cyproheptadine	4–12 mg 1-2 h before sexual activity	Antihistamine with anti-serotonergic properties	Drowsiness, dizziness, dry mouth, constipation, blurred vision, restlessness
Yohimbine	20–40 mg	α-2 antagonist	Tachycardia, anxiety, hypertension, nausea
Buspirone	20–60 mg daily	5-HT1A agonist effect; α-2 antagonist	Dizziness
Bethanechol	10–20 mg 1–2 h before sexual activity or 30–100 mg daily	Muscarinic agonist	Diarrhea, cramps, diaphoresis

*CNS* central nervous system, *NMDA* N-methyl-D-aspartate.

Cabergoline and bupropion are most commonly described in the literature. Cabergoline acts as a dopamine agonist at D2 receptors, resulting in decreased PRL levels. Dopamine has been observed to enhance sexual drive and orgasmic quality and facilitate penile erection, likely by increasing oxytocin release [13, 64, 65]. Acute decreases in PRL levels induced by dopamine agonists may also contribute to enhancing sexual function [66]. In a single-blind balanced crossover study of 10 healthy males, PRL levels were pharmacologically increased; following administration of cabergoline to decrease PRL, sexual drive and function significantly increased [66]. Similarly, a retrospective review of 131 men treated with cabergoline 0.5 mg twice a week at a single andrology clinic for DO/AO demonstrated that 66.7% (*n* = 87) of men reported subjective improvement in orgasm following therapy [67]. Duration of therapy (*p* = 0.03) and concomitant testosterone therapy (*p* = 0.02) were significantly associated with positive response to cabergoline treatment [67]. Furthermore, cabergoline was efficacious regardless of underlying etiology of DO. These studies suggest that acute PRL reduction modulates sexual function and cabergoline may serve as an effective treatment for DO. Prospective randomized trials are still needed.

Bupropion is an atypical antidepressant that inhibits reuptake of dopamine and norepinephrine. Compared to other antidepressants, bupropion has been shown to have lower rates of adverse sexual side effects and may even enhance sexual function in some patients [68]. In rats, bupropion increases contractile response to nerve stimulation in the vas deferens and the epididymal duct [69]. Modell et al. surveyed 107 patients on antidepressants and found that 77% of patients treated with bupropion reported improvement in at least one aspect of sexual functioning [68]. It has been posited that these pro-sexual effects are simply a result of the drug’s antidepressant activity in depressed patients; however, a study of 10 nondepressed men with DO showed that bupropion treatment resulted in significant improvement in sexual satisfaction, ability to achieve erection, and time to reaching orgasm, suggesting pro-sexual effects beyond its antidepressant activity [70]. Likewise, Adbel-Hamid et al. demonstrated a 25% decrease in mean ejaculation latency time and significantly improved orgasm and intercourse satisfaction among 19 men with DO who received bupropion-SR 150 mg daily for 2 months [71].

Oxytocin, a peptide released by the posterior pituitary gland, has been implicated in sexual function [72]. Oxytocin receptors have been identified throughout the male genital tract [73, 74]. Surges of oxytocin have also been observed during ejaculation and suppression of systemic oxytocin release with naloxone has resulted in decreased arousal and pleasure at orgasm [75–77]. Oxytocin has also been implicated in the contraction of the prostatic urethra, ejaculatory duct, and bladder neck [78]. There have been case reports of successful treatment of AO and improvement of sexual function with administration of intranasal oxytocin [79, 80]. Conversely, a prospective randomized study of 102 healthy men showed no significant difference in mean time to ejaculation between men who received intranasal oxytocin (*n* = 49, 10.24 min) compared to the control group (*n* = 53, 10.74 min; *p* = 0.53) [81]. Overall, the existing literature does not show conclusive evidence for the effects of oxytocin administration on sexual function and further studies must be done to clarify this relationship.

Some men with DO experience wandering thoughts during sexual activity and have difficulty concentrating, resulting in diminished sexual arousal. Thus, amphetamine/dextroamphetamine (Adderall^TM^), used in attention-deficit/hyperactivity disorder (ADHD), was trialed as treatment for DO [82]. Eight of 17 men with DO who were treated with Adderall reported improved sexual experience and 6 men experienced reduced orgasmic latency time (OLT). Among those with DO who saw improvement, mean OLT decreased by 72.3%. A case report showed that another ADHD drug, lisdexamfetamine dimesylate (Vyvanse^TM^), was administered to a patient with neurogenic anejaculation and resulted in achievement of ejaculation with masturbation [83]. Additional evidence must be gathered to establish the effectiveness of these medications in DO.

Alpha-1 adrenergic receptors have been identified in the human vas deferens, seminal vesicle, and urethra [84–86]. Alpha-adrenergic receptor agonists including midodrine, imipramine, pseudoephedrine, and ephedrine have been used as treatments for DO. A systematic review of alpha-agonists showed that midodrine had the best rates of anejaculation reversal and the overall success rate of alpha-agonist treatment was 21% [87]. A study of midodrine in 185 men with spinal cord injury and anejaculation found that 64.6% of participants achieved either retrograde or antegrade ejaculation [88]. Large double-blind placebo-controlled studies are needed to truly evaluate these treatments for DO/AO.

There have been small trials using amantadine, cyproheptadine, yohimbine, buspirone, and bethanechol to treat antidepressant-induced sexual dysfunction. Shrivastava et al. successfully used amantadine to treat 6 men with DO secondary to paroxetine [89]. There are multiple case reports using cyproheptadine to treat DO or AO induced by antidepressants [90–92]. A clinical trial of 29 men with orgasmic dysfunction showed successful treatment with yohimbine, a selective competitive alpha-2 adrenergic receptor blocker, in 55% of men [93]. A clinical trial examining the effect of buspirone on sexual dysfunction in patients with depression found that of the 12 men who reported orgasm dysfunction at baseline, about half reported improved orgasm after treatment with buspirone [94]. Bethanechol, a muscarinic receptor agonist, has also been studied. A randomized, double-blind, placebo-controlled crossover trial of 12 men showed improvement in DO induced by antidepressants [95]. A larger sample size is needed to validate the effects of bethanechol in DO or AO. Additionally, more data must be gathered on the application of these medications to sexual dysfunction not induced by antidepressants. The existing literature provides insufficient evidence to recommend the use of these drugs in treatment of DO at this time.

### Choosing the “Right” pharmacotherapy

A variety of oral pharmacotherapies have been studied for DO/AO, however as noted above most of the evidence is derived from small cohorts with incomplete characterization of other confounding factors. Choosing the right oral pharmacotherapy for a patient after addressing organic causes and considering sex therapy can be challenging. The specific treatment should be individualized and guided by an informed patient-provider discussion regarding outcomes and adverse effects. Other considerations include patient preferences such as the dosing regimen (on demand versus scheduled), cost and availability of the medication, and the provider’s level of comfort prescribing a specific medication and monitoring for side effects. The suspected etiology of DO/AO can also help guide treatment. Based on the available evidence, there is no “right” pharmacotherapy, and it is important to have an informed discussion and tailor an individualized treatment plan for men with DO/AO.

### Invasive, non-pharmacological therapies

Some more invasive non-pharmacological therapies have been explored including intracavernosal injections of platelet rich plasma, pudendal nerves release, and surgical procedures. Given the lack of validated studies demonstrating their effectiveness and significant risks that come with invasive procedures, these interventions are not currently recommended as forms of treatment for DO/AO [6].

## Conclusions

Orgasm and ejaculation represent fundamental aspects of male sexual function and disorders affecting these processes garner a tremendous impact on quality of life. DO and AO are prevalent among men and may be underreported due to patient embarrassment or discomfort. Given the complex and multifactorial nature of this condition, the most critical component of patient evaluation is gathering a thorough medical, surgical, and psychosocial history and performing a complete physical examination. Treatment options include addressing underlying organic causes and sex therapy. Though many promising pharmacotherapies have emerged, none are currently approved by the FDA for this indication. Future directions include developing a universal definition of DO and AO as well as a standardized pathway to achieve objective evaluation and diagnosis. Further studies elucidating the underlying molecular pathogenesis of orgasmic dysfunction can also lead to novel targeted pharmacotherapies.

## Data Availability

Data sharing not applicable to this article as no datasets were generated or analysed during the current study.

## References

[1] Nimbi FM, Tripodi F, Rossi R, Navarro-Cremades F, Simonelli C (2020). Male sexual desire: an overview of biological, psychological, sexual, relational, and cultural factors influencing desire. Sex Med Rev.

[2] Aboseif SR, Breza J, Orvis BR, Lue TF, Tanagho EA (1989). Erectile response to acute and chronic occlusion of the internal pudendal and penile arteries. J Urol.

[3] Salonia A, Bettocchi C, Boeri L, Capogrosso P, Carvalho J, Cilesiz NC (2021). European Association of Urology Guidelines on Sexual and Reproductive Health-2021 Update: Male Sexual Dysfunction. Eur Urol.

[4] Rambhatla A, Rajfer J. Male sexual dysfunction. In: Encyclopedia of Endocrine Diseases (Second Edition). 2018. p. 767–74.

[5] Burnett AL, Nehra A, Breau RH, Culkin DJ, Faraday MM, Hakim LS (2018). Erectile dysfunction: AUA Guideline. J Urol.

[6] Shindel AW, Althof SE, Carrier S, Chou R, McMahon CG, Mulhall JP (2022). Disorders of ejaculation: An AUA/SMSNA Guideline. J Urol.

[7] Serefoglu EC, Yaman O, Cayan S, Asci R, Orhan I, Usta MF (2011). Prevalence of the complaint of ejaculating prematurely and the four premature ejaculation syndromes: results from the Turkish Society of Andrology Sexual Health Survey. J Sex Med.

[8] Gao J, Zhang X, Su P, Liu J, Xia L, Yang J (2013). Prevalence and factors associated with the complaint of premature ejaculation and the four premature ejaculation syndromes: a large observational study in China. J Sex Med.

[9] Lew-Starowicz Z, Czajkowska K (2022). Prevalence of sexual dysfunctions and associated risk factors in Poland. Arch Med Sci.

[10] Corona G, Jannini EA, Lotti F, Boddi V, De Vita G, Forti G (2011). Premature and delayed ejaculation: two ends of a single continuum influenced by hormonal milieu. Int J Androl.

[11] American Psychiatric Association. Diagnostic and statistical manual of mental disorders: DSM-5. 2013.

[12] Giuliano F, Clément P (2005). Physiology of ejaculation: Emphasis on serotonergic control. Eur Urol.

[13] Alwaal A, Breyer BN, Lue TF (2015). Normal male sexual function: emphasis on orgasm and ejaculation. Fertil Steril.

[14] Giuliano F (2011). Neurophysiology of erection and ejaculation. J Sex Med.

[15] Gerstenberg TC, Levin RJ, Wagner G (1990). Erection and ejaculation in man. Assessment of the electromyographic activity of the bulbocavernosus and ischiocavernosus muscles. Br J Urol.

[16] Bohlen JG, Held JP, Sanderson MO (1980). The male orgasm: pelvic contractions measured by anal probe. Arch Sex Behav.

[17] Shafik A (1998). The mechanism of ejaculation: the glans-vasal and urethromuscular reflexes. Arch Androl.

[18] Stoléru S, Fonteille V, Cornélis C, Joyal C, Moulier V (2012). Functional neuroimaging studies of sexual arousal and orgasm in healthy men and women: a review and meta-analysis. Neurosci Biobehav Rev.

[19] Holstege G, Georgiadis JR, Paans AMJ, Meiners LC, van der Graaf FHCE, Reinders AATS (2003). Brain activation during human male ejaculation. J Neurosci.

[20] Flannigan R, Heier L, Voss H, Chazen JL, Paduch DA (2019). Functional magnetic resonance imaging detects between-group differences in neural activation among men with delayed orgasm compared with normal controls: Preliminary report. J Sex Med.

[21] World Health Organization. International Classification of Diseases and Related Health Problems 11th Revision. 2018.

[22] Jenkins LC, Mulhall JP (2015). Delayed orgasm and anorgasmia. Fertil Steril.

[23] Montorsi F, Adaikan G, Becher E, Giuliano F, Khoury S, Lue TF (2010). Summary of the recommendations on sexual dysfunctions in men. J Sex Med.

[24] Waldinger MD, Quinn P, Dilleen M, Mundayat R, Schweitzer DH, Boolell M (2005). A multinational population survey of intravaginal ejaculation latency time. J Sex Med.

[25] Kinsey AC, Pomeroy WR, Martin CE (2003). Sexual behavior in the human male. 1948. Am J Public Health.

[26] Nathan SG (1986). The epidemiology of the DSM-III psychosexual dysfunctions. J Sex Marital Ther.

[27] Corona G, Jannini EA, Mannucci E, Fisher AD, Lotti F, Petrone L (2008). Different testosterone levels are associated with ejaculatory dysfunction. J Sex Med.

[28] Corona G, Rastrelli G, Maseroli E, Forti G, Maggi M (2013). Sexual function of the ageing male. Best Pract Res Clin Endocrinol Metab.

[29] Teloken P, Nelson C, Mulhall J. 1384 Secondary delayed orgasm: Patterns, correlates and predictors. J Urol. 2012;187.e562.

[30] Corona G, Mannucci E, Fisher AD, Lotti F, Ricca V, Balercia G (2007). Effect of hyperprolactinemia in male patients consulting for sexual dysfunction. J Sex Med.

[31] Carani C, Isidori AM, Granata A, Carosa E, Maggi M, Lenzi A (2005). Multicenter study on the prevalence of sexual symptoms in male hypo- and hyperthyroid patients. J Clin Endocrinol Metab.

[32] Werneke U, Northey S, Bhugra D (2006). Antidepressants and sexual dysfunction. Acta Psychiatr Scand.

[33] AlBreiki M, AlMaqbali M, AlRisi K, AlSinawi H, Al Balushi M, Al (2020). Prevalence of antidepressant-induced sexual dysfunction among psychiatric outpatients attending a tertiary care hospital. Neuroscience.

[34] Higgins A, Nash M, Lynch AM (2010). Antidepressant-associated sexual dysfunction: impact, effects, and treatment. Drug Health Patient Saf.

[35] Serretti A, Chiesa A (2011). A meta-analysis of sexual dysfunction in psychiatric patients taking antipsychotics. Int Clin Psychopharmacol.

[36] Clayton A, Kornstein S, Prakash A, Mallinckrodt C, Wohlreich M (2007). Changes in sexual functioning associated with duloxetine, escitalopram, and placebo in the treatment of patients with major depressive disorder. J Sex Med.

[37] Grover S, Mattoo SK, Pendharkar S, Kandappan V (2014). Sexual dysfunction in patients with alcohol and opioid dependence. Indian J Psychol Med.

[38] Brown R, Balousek S, Mundt M, Fleming M (2005). Methadone maintenance and male sexual dysfunction. J Addict Dis.

[39] Van de Kar LD, Bethea CL (1982). Pharmacological evidence that serotonergic stimulation of prolactin secretion is mediated via the dorsal raphe nucleus. Neuroendocrinology.

[40] Corona G, Ricca V, Bandini E, Mannucci E, Lotti F, Boddi V (2009). Selective serotonin reuptake inhibitor-induced sexual dysfunction. J Sex Med.

[41] Clayton AH, Croft HA, Horrigan JP, Wightman DS, Krishen A, Richard NE (2006). Bupropion extended release compared with escitalopram: effects on sexual functioning and antidepressant efficacy in 2 randomized, double-blind, placebo-controlled studies. J Clin Psychiatry.

[42] Shull GR, Sprenkle DH (1980). Retarded ejaculation reconceptualization and implications for treatment. J Sex Marital Ther.

[43] Waldinger MD, Schweitzer DH (2005). Retarded ejaculation in men: an overview of psychological and neurobiological insights. World J Urol.

[44] Byun JS, Lyu SW, Seok HH, Kim WJ, Shim SH, Bak CW (2013). Sexual dysfunctions induced by stress of timed intercourse and medical treatment. BJU Int.

[45] Xia J-D, Han Y-F, Pan F, Zhou L-H, Chen Y, Dai Y-T (2013). Clinical characteristics and penile afferent neuronal function in patients with primary delayed ejaculation. Andrology.

[46] Rowland DL (1998). Penile sensitivity in men: a composite of recent findings. Urology.

[47] Haney NM, Alzweri LM, Hellstrom WJG (2018). Male orgasmic dysfunction post-radical pelvic surgery. Sex Med Rev.

[48] Tewari A, Grover S, Sooriakumaran P, Srivastava A, Rao S, Gupta A (2012). Nerve sparing can preserve orgasmic function in most men after robotic-assisted laparoscopic radical prostatectomy. BJU Int.

[49] Bhambhvani HP, Kasman AM, Zhang CA, Hu SS, Eisenberg ML (2022). Delayed ejaculation after lumbar spine surgery: a claims database analysis. Global Spine J.

[50] Abdel-Hamid IA, Ali OI (2018). Delayed ejaculation: pathophysiology, diagnosis, and treatment. World J Mens Health.

[51] Kouidrat Y, Pizzol D, Cosco T, Thompson T, Carnaghi M, Bertoldo A (2017). High prevalence of erectile dysfunction in diabetes: a systematic review and meta-analysis of 145 studies. Diabet Med.

[52] O’Connor DB, Lee DM, Corona G, Forti G, Tajar A, O’Neill TW (2011). The relationships between sex hormones and sexual function in middle-aged and older European men. J Clin Endocrinol Metab.

[53] Morgentaler A, Polzer P, Althof S, Bolyakov A, Donatucci C, Ni X (2017). Delayed ejaculation and associated complaints: relationship to ejaculation times and serum testosterone levels. J Sex Med.

[54] Corona G, Giorda CB, Cucinotta D, Guida P, Nada E (2014). Gruppo di studio SUBITO-DE. Sexual dysfunction at the onset of type 2 diabetes: the interplay of depression, hormonal and cardiovascular factors. J Sex Med.

[55] Jeffries WL, Zsembik BA, Peek CW, Uphold CR (2009). A longitudinal analysis of sociodemographic and health correlates of sexual health among HIV-infected men in the USA. Sex Health.

[56] Gray M, Zillioux J, Khourdaji I, Smith RP (2018). Contemporary management of ejaculatory dysfunction. Transl Androl Urol.

[57] Martin-Tuite P, Shindel AW (2020). Management options for premature ejaculation and delayed ejaculation in men. Sex Med Rev.

[58] Mulhall JP, Trost LW, Brannigan RE, Kurtz EG, Redmon JB, Chiles KA (2018). Evaluation and management of testosterone deficiency: AUA Guideline. J Urol.

[59] Althof SE (2012). Psychological interventions for delayed ejaculation/orgasm. Int J Impot Res.

[60] Abdo CHN (2016). The impact of ejaculatory dysfunction upon the sufferer and his partner. Transl Androl Urol.

[61] Friedman M (1973). Success phobia and retarded ejaculation. Am J Psychother.

[62] Perelman MA, Rowland DL (2006). Retarded ejaculation. World J Urol.

[63] Nelson CJ, Ahmed A, Valenzuela R, Parker M, Mulhall JP (2007). Assessment of penile vibratory stimulation as a management strategy in men with secondary retarded orgasm. Urology.

[64] Krüger THC, Hartmann U, Schedlowski M (2005). Prolactinergic and dopaminergic mechanisms underlying sexual arousal and orgasm in humans. World J Urol.

[65] Danjou P, Lacomblez L, Warot D, Puech AJ (1989). Assessment of erectogenic drugs by numeric plethysmography. J Pharm Methods.

[66] Krüger THC, Haake P, Haverkamp J, Krämer M, Exton MS, Saller B (2003). Effects of acute prolactin manipulation on sexual drive and function in males. J Endocrinol.

[67] Hollander AB, Pastuszak AW, Hsieh T-C, Johnson WG, Scovell JM, Mai CK (2016). Cabergoline in the treatment of male orgasmic disorder-a retrospective pilot analysis. Sex Med.

[68] Modell JG, Katholi CR, Modell JD, DePalma RL (1997). Comparative sexual side effects of bupropion, fluoxetine, paroxetine, and sertraline. Clin Pharmacol Ther.

[69] Cavariani MM, de Almeida Kiguti LR, de Lima Rosa J, de Araújo Leite GA, Silva PVE, Pupo AS (2015). Bupropion treatment increases epididymal contractility and impairs sperm quality with no effects on the epididymal sperm transit time of male rats. J Appl Toxicol.

[70] Modell JG, May RS, Katholi CR. Effect of bupropion-SR on orgasmic dysfunction in nondepressed subjects: a pilot study. J Sex Marital Ther. 2000;26:231–40. 10.1080/00926230050084623 10929571

[71] Abdel-Hamid IA, Saleh E-S (2011). Primary lifelong delayed ejaculation: characteristics and response to bupropion. J Sex Med.

[72] Abdel-Hamid IA, Elsaied MA, Mostafa T (2016). The drug treatment of delayed ejaculation. Transl Androl Urol.

[73] Filippi S, Vannelli GB, Granchi S, Luconi M, Crescioli C, Mancina R (2002). Identification, localization and functional activity of oxytocin receptors in epididymis. Mol Cell Endocrinol.

[74] Frayne J, Nicholson HD (1998). Localization of oxytocin receptors in the human and macaque monkey male reproductive tracts: evidence for a physiological role of oxytocin in the male. Mol Hum Reprod.

[75] Murphy MR, Seckl JR, Burton S, Checkley SA, Lightman SL (1987). Changes in oxytocin and vasopressin secretion during sexual activity in men. J Clin Endocrinol Metab.

[76] Carmichael MS, Humbert R, Dixen J, Palmisano G, Greenleaf W, Davidson JM (1987). Plasma oxytocin increases in the human sexual response. J Clin Endocrinol Metab.

[77] Murphy MR, Checkley SA, Seckl JR, Lightman SL (1990). Naloxone inhibits oxytocin release at orgasm in man. J Clin Endocrinol Metab.

[78] Thackare H, Nicholson HD, Whittington K. Oxytocin–its role in male reproduction and new potential therapeutic uses. Hum Reprod Update. 2006;12:437–48. 10.1093/humupd/dmk002 16436468

[79] IsHak WW, Berman DS, Peters A (2008). Male anorgasmia treated with oxytocin. J Sex Med.

[80] MacDonald K, Feifel D (2012). Dramatic improvement in sexual function induced by intranasal oxytocin. J Sex Med.

[81] Walch K, Eder R, Schindler A, Feichtinger W (2001). The effect of single-dose oxytocin application on time to ejaculation and seminal parameters in men. J Assist Reprod Genet.

[82] Levine LA, Betcher HK, Ziegelmann MJ, Bajic P (2020). Amphetamine/Dextroamphetamine salts for delayed orgasm and anorgasmia in men: a pilot study. Urology.

[83] Lyons MD, Lentz AC, Coward RM (2017). Lisdexamfetamine Dimesylate (Vyvanse) for the treatment of neurogenic anejaculation. Am J Mens Health.

[84] Hedlund H, Andersson KE, Larsson B (1985). Effect of drugs interacting with adrenoreceptors and muscarinic receptors in the epididymal and prostatic parts of the human isolated vas deferens. J Auton Pharmacol.

[85] de Almeida Kiguti LR, Pupo AS (2012). Investigation of the effects of α1-adrenoceptor antagonism and L-type calcium channel blockade on ejaculation and vas deferens and seminal vesicle contractility in vitro. J Sex Med.

[86] Hisasue S, Furuya R, Itoh N, Kobayashi K, Furuya S, Tsukamoto T (2006). Ejaculatory disorder caused by alpha-1 adrenoceptor antagonists is not retrograde ejaculation but a loss of seminal emission. Int J Urol.

[87] Kamischke A, Nieschlag E (2002). Update on medical treatment of ejaculatory disorders. Int J Androl.

[88] Soler JM, Previnaire JG, Plante P, Denys P, Chartier-Kastler E (2007). Midodrine improves ejaculation in spinal cord injured men. J Urol.

[89] Shrivastava RK, Shrivastava S, Overweg N, Schmitt M (1995). Amantadine in the treatment of sexual dysfunction associated with selective serotonin reuptake inhibitors. J Clin Psychopharmacol.

[90] Riley AJ, Riley EJ (1986). Cyproheptadine and antidepressant-induced anorgasmia. Br J Psychiatry.

[91] Arnott S, Nutt D (1994). Successful treatment of fluvoxamine-induced anorgasmia by cyproheptadine. Br J Psychiatry.

[92] Keller Ashton A, Hamer R, Rosen RC (1997). Serotonin reuptake inhibitor-induced sexual dysfunction and its treatment: a large-scale retrospective study of 596 psychiatric outpatients. J Sex Marital Ther.

[93] Adeniyi AA, Brindley GS, Pryor JP, Ralph DJ (2007). Yohimbine in the treatment of orgasmic dysfunction. Asian J Androl.

[94] Landén M, Eriksson E, Agren H, Fahlén T (1999). Effect of buspirone on sexual dysfunction in depressed patients treated with selective serotonin reuptake inhibitors. J Clin Psychopharmacol.

[95] Bernik M, Vieira AHG, Nunes PV (2004). Bethanecol chloride for treatment of clomipramine-induced orgasmic dysfunction in males. Rev Hosp Clin Fac Med Sao Paulo.

